# Exome Sequencing from Nanogram Amounts of Starting DNA: Comparing Three Approaches

**DOI:** 10.1371/journal.pone.0101154

**Published:** 2014-07-03

**Authors:** Vera N. Rykalina, Alexey A. Shadrin, Vyacheslav S. Amstislavskiy, Evgeny I. Rogaev, Hans Lehrach, Tatiana A. Borodina

**Affiliations:** 1 Max-Planck Institute for Molecular Genetics, Berlin, Germany; 2 AlacrisTheranostics GmbH, Berlin, Germany; 3 Freie Universität Berlin, Berlin, Germany; 4 Vavilov Institute of General Genetics, Moscow, Russia; Natural History Museum of Denmark, Denmark

## Abstract

Hybridization-based target enrichment protocols require relatively large starting amounts of genomic DNA, which is not always available. Here, we tested three approaches to pre-capture library preparation starting from 10 ng of genomic DNA: (i and ii) whole-genome amplification of DNA samples with REPLI-g (Qiagen) and GenomePlex (Sigma) kits followed by standard library preparation, and (iii) library construction with a low input oriented ThruPLEX kit (Rubicon Genomics). Exome capture with Agilent SureSelect*^XT2^* Human AllExon v4+UTRs capture probes, and HiSeq2000 sequencing were performed for test libraries along with the control library prepared from 1 µg of starting DNA. Tested protocols were characterized in terms of mapping efficiency, enrichment ratio, coverage of the target region, and reliability of SNP genotyping. REPLI-g- and ThruPLEX-FD-based protocols seem to be adequate solutions for exome sequencing of low input samples.

## Introduction

Whole exome sequencing (WES) is currently one of the main applications in next generation sequencing and this trend will likely continue in the near future. WES requires much less sequencing volume, allows higher throughput, and requires less computational resources than whole genome sequencing (WGS). WES is about ten times cheaper than WGS (calculated for the following WES settings: library preparation with Agilent SureSelect Human All Exon v4 kit, sequencing on Illumina HiSeq2000 platform in paired end 2×100 bp sequencing mode, with 12 GB output of filtered data; and the following WGS settings: library preparation with Illumina TruSeq library preparation kit, sequencing on Illumina HiSeq2000 platform in paired end 2×100 bp sequencing mode, with 150 GB output of filtered data). In many applications these advantages make WES a feasible alternative to WGS in terms of the price/results ratio. Multiple publications have demonstrated the impact of WES in identifying causative variants of Mendelian diseases [1]–[7]. WES is also performed to analyze complex traits, to both reveal trait-associated regions and screen for individual variations contributing to the trait manifestation [8]–[10].

Currently the preferred method for WES library preparation is hybridization-based enrichment of whole genome sequencing libraries [11]. Corresponding commercial products are available for example from Agilent, NimbleGen, and Illumina. Existing exome enrichment kits differ in total size of target region, and the number, length and nature (DNA or RNA) of the capture probes, as well as minor issues in laboratory procedures; however the principle of the protocol is the same. The procedure begins with preparing a whole genome library – random genomic DNA fragments flanked with common adapters. The library is amplified with several PCR cycles and mixed with a set of artificial biotinilated probes corresponding to the target region. Library molecules with inserts at least partly containing fragments of the target region hybridize to the capture probes. Capture probes, both free and hybridized to library molecules, are collected by their biotin groups using streptavidin-coated magnetic beads. Library molecules are then washed off the beads and amplified to a concentration appropriate for sequencing.

Hybridization-based exome enrichment kits require comparatively large amount of starting genomic DNA, i.e. 1–3 µg. For comparison, a WGS library of good complexity may be prepared from just 10 ng of genomic DNA. However, in many cases even such amounts are not available, for example in small size samples such as clinical biopsies. Another example is DNA collections in population analysis and genome-wide associated studies (GWAS) laboratories. Researchers try to use the material carefully, since it is hard to assemble such collections and samples may be required for several projects.

Cost-efficiency of exome sequencing makes it very attractive to be applied for low quantity samples. Several approaches have been suggested to overcome the sample amount requirement and a number of publications describe exome sequencing performed with sub-microgram amounts of starting DNA.

One approach is to increase the amount of starting material by whole genome amplification. In this case the WES library is prepared from the recommended amount of material and no changes to the protocol itself are necessary [12], [13].

Another approach is to optimise the whole genome library preparation that precedes the hybridization-based enrichment (pre-capture library). The standard procedure involves DNA shearing followed by three enzymatic steps, with two purifications and amplification in between. During library preparation loss of material occurs due to the wide distribution of fragment sizes after DNA shearing, taking aliquots for quality and quantity evaluations, and purification steps. It is possible to reduce these losses and adjust the standard protocol for smaller starting amounts [14].

An alternative transposon-based library preparation method (Nextera technology, Illumina) gives enough material for hybridization-based enrichment staring with just 50 ng of genomic DNA. The efficiency of this method is explained by obtaining fragmented adapter-flanked genomic DNA in just one tagmentation reaction (material losses are minimized) and by a more narrow size distribution of fragments than obtained by e.g. ultrasonic shearing. However this method is sensitive to the fragmentation of starting DNA and produces more biased coverage of genomic regions [15].

The amount of input material for hybridization-based enrichment steps can also be reduced, as described in the MSA-Cap method suggested by Kosarewa et al. [14]. Except for optimising the steps prior to hybridization, the authors used the fact that commercial protocols are not working at the border of sensitivity; they used half the recommended amount of library for hybridization. In addition, they optimised the post hybridization procedure, decreasing the concentration of the library solution required for Illumina sequencing. All together, they managed to decrease the starting amount for exome sequencing using the Agilent SureSelect capture method from 3 µg to 50 ng.

During the ADAMS FP7 EU project we faced the necessity of performing exome sequencing and target sequencing of GWAS selected regions with low amounts of samples. Our partners in the ADAMS consortium have large DNA collections, often stored for a long time, of different quality. We had to look for a strategy to prepare libraries for exome sequencing starting from about 10 ng of genomic DNA.

To prepare the WES library we tested three commercially available systems for pre-capture library preparation: REPLI-g (Qiagen), GenomePlex (Sigma) and ThruPLEX-FD (Rubicon Genomics). Exome enrichment was performed with Agilent SureSelect*^XT2^* Human AllExon v4+UTRs capture probes sets. Sequencing was performed on the Illumina HiSeq2000 platform. We compared three test sequencing data sets with data obtained from the recommended starting amount of material, using evaluation parameters commonly used to characterize exome sequencing: mapping efficiency, enrichment efficiency, coverage uniformity, and single nucleotide variants (SNV) detection efficiency.

## Materials and Methods

### DNA samples

Protocols compared in this study were independently tested on two human genomic DNA samples. Test DNA 1 was purchased from Bioline (Human Genomic DNA, BIO-35025). Test DNA 2 was isolated from peripheral blood of an anonymous blood donor using phenol-chloroform method. Original DNA purity and integrity was confirmed by gel electrophoresis. Concentrations were determined on a Qubit fluorometer using a dsDNA BR kit (Invitrogen, Q32853).

The blood sample for Test DNA 2 used for this work is one of the samples collected specifically for the ADAMS FP7 project, mentioned in the Funding section. All samples for this project were taken with written informed consent and all the data was anonymized. This particular sample comes from the group of one of the co-authors, Prof. Evgeny Rogaev. Prof. Rogaev got the approval of the Local Ethical Committee of Vavilov Institute of General Genetics of Russian Academy of Sciences for the ADAMS FP7 project. Prof. Rogaev did not collect blood himself and did not contact the donor, but he has access to the donor-identifying information.

### Whole genome amplification

Whole genome amplification (WGA) was performed using the commercially available GenomePlex Complete Whole Genome Amplification Kit (Sigma, Cat. No. WGA2) and REPLI-g Mini Kit (Qiagen, Cat. No. 150023) following the manufacturers’ protocols. Starting amounts of genomic DNA for WGA reactions was 10 ng in all cases.

The **REPLI-g** protocol includes denaturation of DNA and isothermal amplification. About 15 minutes hands-on time are required at the beginning. Total procedure time is determined by the recommended amplification duration: 10–16 hours.

DNA was first denatured: 5 µl of Buffer D1 were added to 5 µl of 2 ng/µl DNA solution and the sample was incubated at room temperature for 3 minutes. Denaturation was stopped by adding 10 µl of neutralization buffer N1. Then 30 µl of REPLI-g Master Mix were added to the sample, and amplification was carried out at 30°C for 16.5 hours. REPLI-g Mini DNA Polymerase was inactivated by heating at 65°C for 3 minutes.

The **GenomePlex** WGA protocol includes (i) fragmentation, (ii) preparation of OmniPlex library and (iii) amplification steps. No intermediate purifications are required. The procedure takes about 5 hours and requires about 30 minutes hands-on time. DNA was first fragmented: 1 µl of 10x Fragmentation Buffer was added to 10 µl of 1 ng/µl DNA solution and the sample was incubated at 95°C for 4 minutes and immediately cooled on ice.

Fragmented DNA was converted into OmniPlex library, fragments flanked with common sequences. First, 2 µl of 1x Library Preparation Buffer and 1 µl of Library Stabilization Buffer were added to the sample after fragmentation. The tube was incubated at 95°C for 2 minutes and cooled on ice. Then 1 µl of Library Preparation Enzyme was added and the sample was placed in a thermal cycler and incubated as follows: 16°C for 20 minutes; 24°C for 20 minutes; 37°C for 20 minutes; 75°C for 5 minutes; 4°C hold.

The OmniPlex library was diluted with 47.5 µl of water, 7.5 µl of 10x Amplification Master Mix and 5 µl of WGA DNA Polymerase and placed in thermal cycler. Following initial denaturation at 95°C for 3 minutes, 14 amplification cycles were performed: 94°C for 15 seconds; 65°C for 5 minutes.

WGA reactions were purified with Agencourt AMPure XP beads (Beckman Coulter, Cat. No. A63881). Concentrations of amplified DNA were measured with Qubit dsDNA BR kit. Both GenomePlex and REPLI-g amplification procedures showed ∼230-fold amplification.

### Pre-capture sample processing

**Figure 1:**
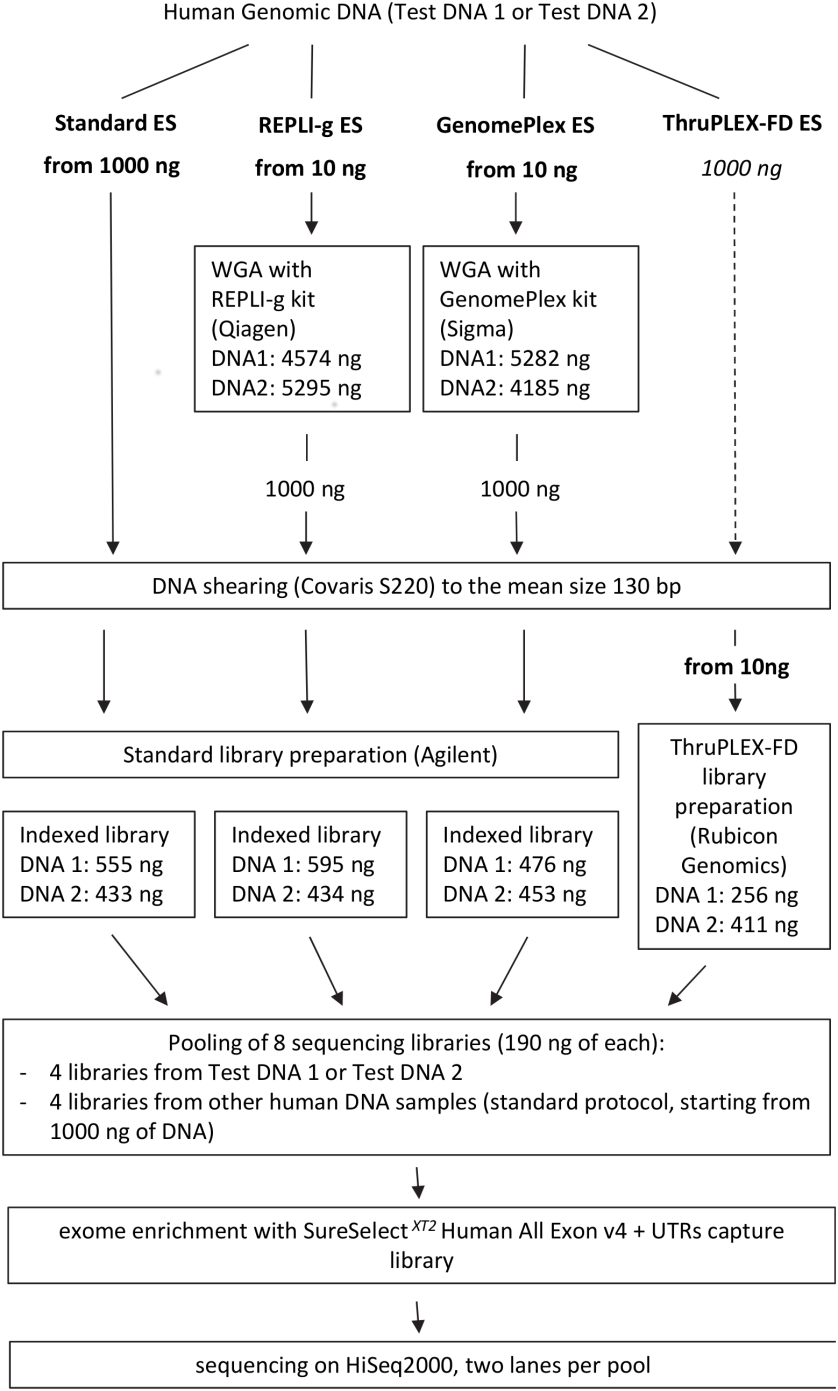
The experimental scheme. Two DNA samples (Test DNA 1 and Test DNA 2) were subjected to four exome sequencing (ES) protocols performed in parallel: control (Standard ES) and three modified (REPLI-g ES, GenomePlex ES and ThruPLEX-FD ES). Common steps performed in parallel for several protocols are shown by text boxes spanning the corresponding number of protocol columns.

For each DNA sample, four barcoded libraries were prepared for further exome capture (Figure 1). Three libraries were prepared starting from 1000 ng of original or WGA-material according to the sample preparation guidelines of the Agilent SureSelect*^XT2^* Target Enrichment System for Illumina Multiplexed Sequencing Protocol (version A, January 2012). One library was prepared starting from 10 ng of sheared original DNA using ThruPLEX Fragmented DNA Prep kit (ThruPLEX-FD) from Rubicon Genomics (Cat. No. R40012).

#### DNA shearing

The first step in both Agilent SureSelect*^XT2^* and ThruPLEX-FD library preparation is DNA fragmentation, which was performed on a Covaris S220 system. Shearing of 1000 ng was carried out in 50 µl of 1x TE buffer using the following instrument parameters: duty cycle 10%, intensity 5, cycles per burst 200, time 6 cycles per 60 seconds. The resulting size distribution was checked using the Agilent 2100 BioAnalyserDNA1000 assay. For all samples, shearing worked very consistently and the size distribution peak was around 130 bp.

In our experience shearing of small amounts of genomic DNA on Covaris S220 is associated with noticeable loss of material. For example, after fragmentation of 20 ng of genomic DNA, only 10 ng are detected. Most probably this is related to the adhesion of DNA molecules to the glass walls of the tubes used for shearing (microTUBE AFA Fiber tubes, Covaris, Cat. No. 520045). To avoid incorrect evaluation of the ThruPLEX-FD kit, we took as starting material 10 ng of already sheared original DNA.

#### Agilent SureSelect*^XT2^* library preparation

Libraries from original, GenomePlex- and REPLI-g- amplified DNA were prepared with SureSelect^XT2^ Reagent kit (Agilent, Cat. No. G9621A) following the manufacturer’s instructions. The procedure comprises standard steps for Illumina DNA library preparation: end-repair of fragmented DNA, A-tailing, adapter ligation and amplification. Purifications between steps were carried out with Agencourt AMPure XP beads. Library preparation takes about 6 hours, and about 2 hours hands-on time. The libraries’ yield, estimated with Qubit dsDNA HS kit (Invitrogen, Q32853), varied between 476 and 595 ng for Test DNA 1 and between 433 and 453 ng for Test DNA2.

#### ThruPLEX-FD library preparation

The ThruPLEX–FD procedure comprises three steps: (i) template preparation, (ii) library synthesis and (iii) amplification and indexing. No intermediate purifications are required; reagents are subsequently added to the same tube. The procedure takes in total 3.5 hours with 45 minutes hands-on time. From 10 ng of starting material we obtained 256 ng for the Test DNA1 library and 411 ng for the Test DNA 2 library (measured with Qubit dsDNA HS kit).

ThruPLEX-FD libraries were prepared following the manufacturer’s protocol. First, 3 µl of Template Preparation pre-mix were added to 10 µl of 1 ng/µl DNA solution and the sample was incubated in a thermal cycler under following conditions: 22°C for 25 minutes; 55°C for 20 minutes, 22°C for 5 minutes. Immediately 2 µl of Library Synthesis pre-mix were added to the tube and the sample was incubated at 22°C for 40 minutes and cooled at 4°C for 5 minutes. Again immediately, 58 µl of Library Amplification pre-mix and 2 µl of one Indexing Reagent (1–12) were added to the sample and the tube was incubated in a thermal cycler using the following amplification program: 72°C for 3 minutes, 85°C for 2 minutes, 98°C for 2 minutes, 4 cycles of (98°C for 20 seconds, 67°C for 20 seconds, 72°C for 40 seconds), 7 cycles of (98°C for 20 seconds, 72°C for 50 seconds). Ready barcoded libraries were purified with Agencourt AMPure XP beads.

### Exome enrichment

Exome capture was performed with Agilent SureSelect*^XT2^* Human All Exon v4 + UTRs capture probes set (Agilent, Cat. No. 5190-4671). The SureSelect*^XT2^* Target Enrichment System is designed for exome capture of eight pooled libraries.

Libraries from Test DNA 1 and Test DNA 2 were prepared and processed within a two-month interval. To prepare library pools for exome enrichment, four test DNA libraries were pooled with four libraries from other human genomic DNA samples. In both cases those were genomic DNA samples isolated from peripheral blood of anonymous donors and converted into libraries with the standard Agilent SureSelect*^XT2^* protocol starting from 1000 ng of DNA. 190 ng of each library were taken for pooling. In both cases the pool volume was larger than the 7 µl required for the following enrichment step, and the pools were concentrated using a SpeedVac concentrator.

Hybridization of pooled libraries to the capture probes and removal of non-hybridized library molecules were carried out according to the Agilent SureSelect^XT2^ Target Enrichment System for Illumina Multiplexed Sequencing Protocol (version A, January 2012).

Library molecules fished out by hybridization were amplified. Excessive amplification can increase sequence bias and cause PCR artefacts, so prior to amplification of the whole sample, a small aliquot (1 µl) of captured library was first tested in qPCR. The number of cycles corresponding to the mid-exponential phase of the amplification curve and adjusted to the volume of the captured library was taken for amplification. For both pools with Test DNA 1 and Test DNA 2 libraries post-hybridization PCR was performed with 11 cycles.

### Sequencing

Sample dilution, flowcell loading and sequencing were performed according to the Illumina specifications. Pools with Test DNA 1 and Test DNA 2 libraries were each sequenced on two lanes of the HiSeq2000 platform as paired-end 101-bp reads.

### Data processing and statistics

All sequencing data are submitted to the European Nucleotide Archive (ENA study accession number PRJEB6077).

Fastq files were generated with Illumina BCL2FASTQ Conversion Software (version 1.8.2).

Some GenomePlex ES library reads contain sequences of the primer used for whole genome amplification. These technical segments were mostly removed before alignment. However since this primer has partly degenerate sequence, it is difficult to completely remove it from the reads.

Bowtie 2 (version 2.1.0) [16] was used to align the reads on human genome reference assembly (build hg19 GRCh37). The genome was downloaded from the UCSC Genome Browser website (http://genome.ucsc.edu/) [17].

The BED file with description of the target region of the SureSelectV4+UTRs kit was provided by the manufacturer. Brief specification of it is shown in Table 1.

**Table 1:** Features of the target region.

Number ofsegments	Total lengthof target region(Mb)	Mean lengthof the segment(bp)	Median lengthof thesegment (bp)	Maximum lengthof the segment(bp)	Minimum lengthof the segment(bp)
199268	70.37	353.1	203	21747	114

After alignment, potential PCR duplicates were removed with Picard MarkDuplicates (version 1.91) [18]. Subsequently, using a home-made script, all reads with a probability of wrong mapping higher than 0.05 according to their mapping quality score (MAPQ<14) were filtered out from each library, the remaining reads were further filtered to remove the ambiguous reads (not uniquely aligned). Then from high-confident uniquely mapped reads, only reads overlapping the target region (TR) were selected with BEDTools (version 2.17.0) [19]. We also estimated the overlapping of high-confident reads with the flanking regions (FR), which include 100 bp from both ends of the targeted sequences.

SNVs and small INDELs were called using SAMtools’ mpileup (version 0.1.19) [20].

## Results

### Strategies for pre-capture library preparation

Our aim was to select a strategy to perform exome sequencing starting from 10 ng of human genomic DNA. For exome capture we chose the Agilent SureSelect*^XT2^* method. The XT2 modification of the SureSelect strategy is designed for hybridization-based enrichment of indexed libraries and seemed attractive in two aspects. First, it allows one to pool up to 8 libraries prior to capture, and is thus cheaper per sample while providing higher throughput. Second, the recommended starting DNA amount is 1 µg and the amount of whole genome library required for hybridization is 190 ng, instead of 3 µg and 500 ng, respectively, for single library processing.

Since hybridization efficiency depends on the concentration of the participating molecules, we wanted to modify the WES library preparation procedure before the hybridization step to obtain the amount of input material for hybridization as recommended by the manufacturer. We selected two approaches: (i) whole genome amplification of initial DNA, to obtain enough input material for a standard Agilent SureSelect protocol, and (ii) preparation of the pre-capture library starting with 10 ng of input material using an optimized protocol, and adopting the Agilent SureSelect protocol at the hybridization step.

For the first approach we chose two commercially available WGA kits: REPLI-g from Qiagen and GenomePlex from Sigma.

The REPLI-g kit uses isothermal genome amplification, called Multiple Displacement Amplification (MDA), which involves random hexamers binding to denatured DNA followed by strand displacement synthesis at a constant temperature using the Phi 29 polymerase [15]. Additional priming events can occur on each displaced strand, leading to a network of branched DNA structures. Phi 29 polymerase does not dissociate from the genomic DNA template, allowing the generation of DNA fragments up to 100 kb without sequence bias.

The GenomePlex Complete Whole Genome Amplification Kit uses the proprietary amplification OmniPlex technology [21]. The protocol involves isothermal primer extension on randomly fragmented genomic DNA. Oligonucleotides for primer extension have self-inert degenerate 3′ ends and universal 5′ tails. Extension is performed with polymerase with strand displacement activity, so, as in MDA amplification, brunched DNA structures are formed. This whole genome amplification step produces multiple, comparatively short fragments with common ends. These fragments are further amplified by PCR amplification using common tails, resulting in DNA fragments with an average size of 400 bp.

Both REPLI-g and GenomePlex technologies were reported to enable accurate genotyping [22], [23], which is also important for targeted sequencing. REPLI-g amplified DNA was also previously used for exome sequencing [12], [13]. GenomePlex has not yet been tried for exome sequencing, and we expected it to perform worse than REPLI-g for target enrichment since it is known that PCR-based WGA is prone to biased representation of different genomic regions. However we decided to compare and characterize this kit, since it is capable of amplifying denatured and degraded DNA. Moreover, if the performance of GenomePlex was acceptable, we knew it would be possible to incorporate NGS adapter sequences during the PCR step of the WGA, and thus simplify library preparation.

For the second approach, we decided to check an alternative library preparation method that had recently appeared on the market: the ThruPLEX-FD Kit from Rubicon Genomics. This kit focuses on NGS library preparation starting from small amounts; enzymatic reactions are optimized and performed in a single tube, without intermediate purifications. Background is minimized, e.g. ligation adapters are destroyed after ligation. The ThruPLEX-FD Kit was also reported to be applicable for degraded DNA samples.

For each of two human genomic DNA samples, Test DNA 1 and Test DNA 2, we performed four protocols of pre-capture library preparation for exome sequencing (Fig. 1): one for recommended starting amounts, and three for 10 ng of initial DNA. Test DNA 1 was high quality placenta DNA (Bioline). Test DNA 2 was DNA isolated from peripheral blood. Both samples were of good quality: RNA-free, and not fragmented.

The standard exome sequencing (ES) protocol was performed completely according to the Agilent recommendations, starting with 1000 ng of initial material and serving as a reference for the modified protocols. REPLI-g ES and GenomePlex ES protocols started with 10 ng of genomic DNA. DNA was first subjected to WGA. 1000 ng of amplified DNA were processed in parallel to the Standard ES sample according to the Agilent library preparation protocol. The ThruPLEX-FD ES protocol started with 10 ng of genomic DNA preliminary sheared to ∼130 bp. 190 ng of amplified library were incorporated into the Agilent protocol starting from the hybridization-based enrichment step. Protocols details, as well as comments on intermediate results, particular protocol characteristics and performance are presented in the Methods section.

Sequencing data from four Test DNA 1 and four Test DNA 2 libraries allowed us to compare low-amount libraries to the recommended input library in the most important quantitative and qualitative parameters: enrichment efficiency, uniformity of coverage of the target region, and accuracy of SNV detection.

### Comparison of sequencing statistics

The alignment statistics for the two sets of libraries grouped by the DNA of origin include basic library properties: mapping efficiency, duplication levels, and enrichment efficiency (Table 2). Generally, there are inter-sample differences, but differences between the library preparation methods are reproduced in both cases. REPLI-g ES library characteristics are very similar to those of the control Standard ES library. GenomePlex ES and ThruPLEX-FD ES libraries demonstrate poorer results.

**Table 2:** Alignment statistics.

LibraryPreparationmethod	Number of rawreads (Mb of seq)	Percentage ofduplicates (% ofraw reads)	Percentage of high-confident* reads mappedto hg19 (% ofraw reads)	Percentage of high-confidentreads mapped uniquelyto hg19 (% of raw reads reads)				Percentage of high-confident readsmapped uniquelyto FR*** (%of raw reads)	Percentage of high-confident readsmapped uniquelyto TR(% of rawreads)
				Total	Mate is mapped(% of total)	Mate is onthe samechromosome (% oftotal)	Mate is onthe samechromosome and hasproperorientation (% of total)		
Standard ES									
Test DNA 1	95033226 (9598)	20.21	75.62	71.82	99.51	99.26	99.25	50.20	47.97
Test DNA 2	173021034 (17475)	25.07	71.31	68.02	99.79	99.73	99.73	49.06	47.34
GenomePlex ES									
Test DNA 1	65957628 (5740)**	18.21	69.54	63.09	96.58	95.94	95.21	31.87	30.40
Test DNA 2	70253046 (6252)**	20.58	63.54	57.49	95.39	94.09	93.58	31.95	31.00
ThruPLEX-FD ES									
Test DNA 1	60302154 (6091)	34.43	61.02	57.83	99.04	97.45	97.41	40.26	38.25
Test DNA 2	81220550 (8203)	45.38	50.10	47.54	98.93	97.08	97.03	31.73	30.28
REPLI-g ES									
Test DNA 1	89106596 (8999)	20.70	75.59	71.90	99.66	99.53	99.53	50.99	49.03
Test DNA 2	146075078 (14754)	25.30	71.64	68.38	99.61	99.54	99.34	49.72	48.04

*high confident reads-reads with probability of wrong mapping lower than 0.05 according to their MAPQ score (MAPQ>13).

**some of GenomePlex ES library reads contained sequences of the primer used for whole genome amplification. These common segments were cut out before the alignment. As a result, 13.8% of and 11.9% of nucleotides were removed from the reads of the Test DNA 1 and Test DNA 2 libraries, respectively.

***FR-flanking regions (FR), which include 100****bp from both ends of the targeted sequences.

The number of raw reads varies between the libraries. We cannot exclude that this is not related to some preference during hybridization. In our experience when exome enrichment is performed on pooled libraries, low-complexity samples are underrepresented in the final library. Moreover ThruPLEX-FD ES libraries, which have smaller numbers of reads, also show lower complexity. However, libraries prepared by the standard protocol (starting from 1000 ng of genomic DNA) also vary considerably in the number of reads (see Figure S1).

ThruPLEX-FD ES libraries show a considerably higher percentage of duplexes: 34% (45%) for Test DNA 1 (Test DNA 2). In comparison, Standard ES and REPLI-g ES libraries have ∼20.5% (25%) duplexes for test DNAs. The GenomePlex ES library looks slightly better with 18% (21%) duplexes.

GenomePlex ES and especially ThruPLEX-FD ES libraries have noticeably less reads mapped to the genome: 70% (64%) and 61% (50%) versus ∼76% (71.5%) for the other two protocols. The GenomePlex ES library shows about two-fold more non-uniquely mapped reads −6.5% (6%), than the other three protocols: 3.2–3.8% (2.6–3.3%). Also GenomePlex ES libraries have slightly more read pairs with improper position of the mate read.

With ThruPLEX-FD ES and GenomePlex ES libraries less reads mapped uniquely to the target region (TR). However for ThruPLEX-FD ES libraries, uniquely mapped TR reads constitute 66% (64%) of the total uniquely mapped reads, a proportion close to Standard ES (67% (70%)) and REPLI-g ES (68% (70%)) libraries. In contrast, for GenomePlex ES only 48% (54%) of uniquely mapped reads fall into the TR. Thus the enrichment efficiency is noticeably worse for GenomePlex ES libraries.

### Target region coverage

To compare coverage features of the target region, such as evenness and depth of coverage, it is crucial to have the comparable amount of sequencing data (Mb) for each library. The data should also be independent from the total number of reads and the enrichment efficiency. Therefore, we made a sub-selection of high-confident reads uniquely mapped to TR, down-sampled with Picard DownsampleSam [18]. Except for GenomePlex ES libraries, approximately 17×10^6^ reads for each library were extracted. In the GenomePlex ES libraries reads were on average shorter due to the removal of the common primer sequence. Hence we took more reads (approximately 19×10^6^ reads for each GenomePlex ES library), in order to approximately equalize the total number of bases of the extracted sequence among all samples (∼17×10^8^ bases). All the results presented here were obtained for these sets of comparable amounts of sequencing data uniquely mapped to the target region (Table 3).

**Table 3:** Coverage statistics for the target region.

Amplification method	Mean coverage	Coverage depth (% of bases in TR)							
		0	1–10	11–20	21–30	31–40	41–50	51–60	61+
Standard ES									
Test DNA 1	20.80	1.56	30.75	29.95	17.49	9.12	4.70	2.48	3.83
Test DNA 2	20.07	2.46	34.32	26.60	15.65	8.82	4.95	2.81	4.05
GenomePlex ES									
Test DNA 1	19.59	8.85	39.18	21.20	11.53	6.55	3.89	2.44	5.92
Test DNA 2	17.79	11.14	39.75	19.97	10.95	6.25	3.71	2.29	5.06
ThruPLEX-FD ES									
Test DNA 1	19.90	1.27	26.61	32.28	20.85	10.51	4.71	2.06	1.62
Test DNA 2	19.07	1.56	26.95	31.34	21.94	11.57	4.60	1.40	0.41
REPLI-g ES									
Test DNA 1	20.92	1.99	30.96	28.39	17.17	9.48	5.11	2.79	3.98
Test DNA 2	20.01	3.41	36.06	25.67	14.58	8.08	4.56	2.61	4.65

Analysis was performed on subsets of reads uniquely mapped to the target region and having approximately equal total amounts of bases (**∼**17**×**10^8^ bases).

Since we selected approximately equal amounts of sequencing data from each sample, the expected values of mean coverage should be very similar, which what we see in Table 3.

Evenness of coverage is an important characteristic of sequencing data for exome analysis. Finding genetic variations is a common task in exome analysis and the more uniform the coverage, the less amount of sequencing data is required to reliably detect variations and be able to use more straightforward and reliable statistical techniques.

Ideally the target region should be uniformly represented in the sequencing data. However sequence-dependent performance at many stages of the sequencing protocol, related to the composition and structure of the DNA, distort the sequences’ representation in the original DNA [24]. For example, it is known that PCR amplification introduces a bias in standard Illumina library preparation depending on the GC composition of the sequence [25]. Both extremely GC-poor and extremely GC-rich loci are often underrepresented or even absent [26]. In the case of target enrichment, capture preferences add to the sequencing bias [27]. As a result, the region being sequenced will have segments that are either over- or under-represented, or completely absent in the sequencing reads.

**Figure 2:**
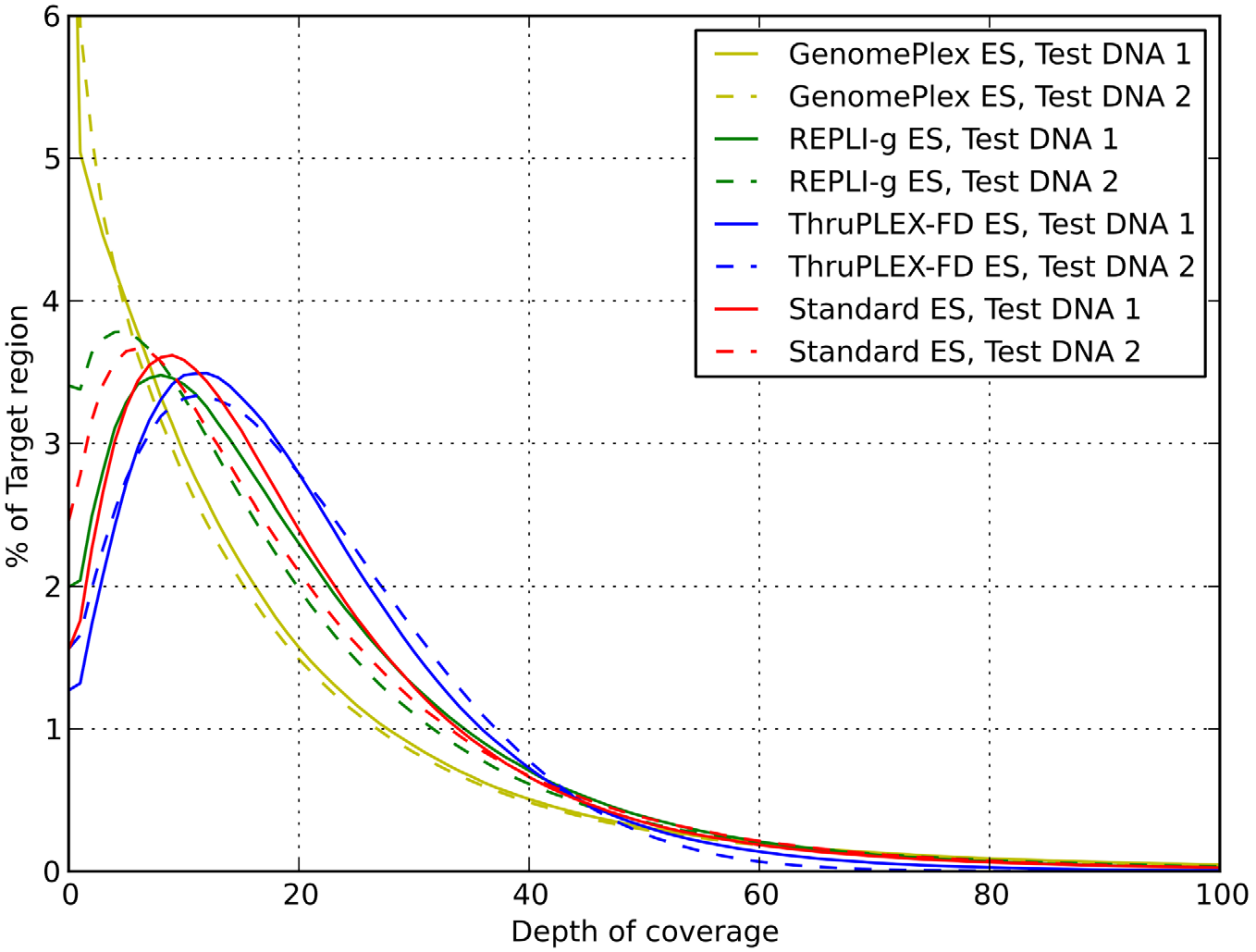
Per-base sequencing depth distribution on the target region.

The uniformity of coverage in the test libraries compared to the standard protocol was assessed and compared (Table 3, Fig. 2). GenomePlex ES libraries differ from the others: 9% (11%) of the TR are not covered at all for Test DNA 1 (Test DNA 2) and only 52% (48%) of the TR have coverage >10. ThruPLEX-FD ES libraries look the best: they demonstrate coverage >10 for 72% (71%) of the TR, while Standard ES libraries have coverage >10 for 68% (63%), and REPLI-g ES libraries for 67% (60%) of the TR.

**Figure 3:**
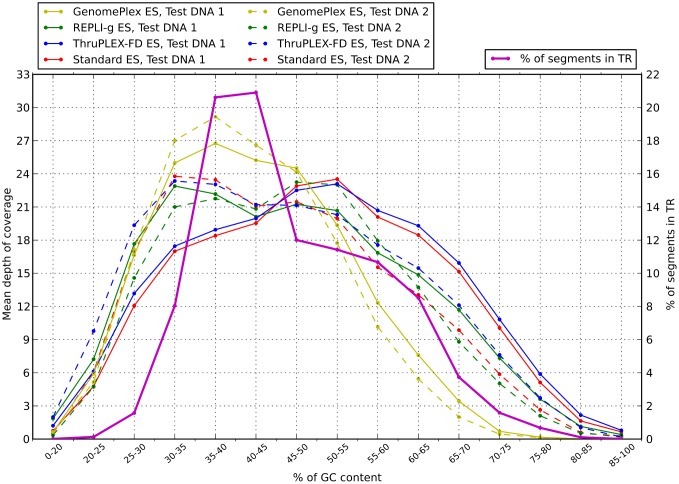
Coverage distribution along the target regions with different percentages of GC bases.

For the mean coverage according to the GC content, ThruPLEX-FD ES libraries look most similar to controls for both DNA samples (Fig. 3). GenomePlex ES libraries show considerable under-representation of sequences with GC content ≥55%. Interestingly, except for GenomePlex ES libraries, all libraries show inter-sample differences in GC content profile.

**Figure 4:**
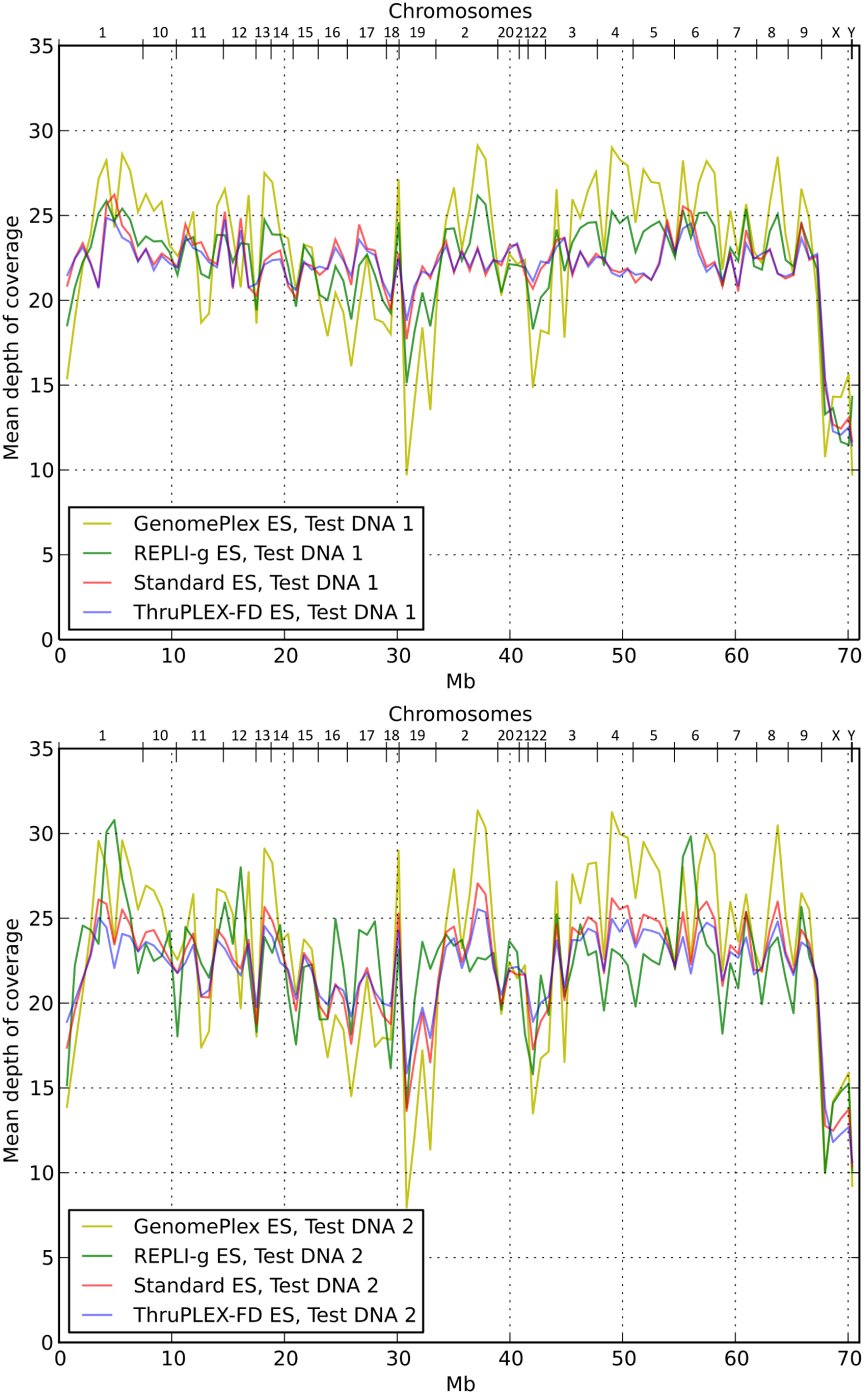
Profiles of coverage depth along the target region for Test DNA 1 (upper panel) and Test DNA 2 (lower panel) WES libraries.

We next asked whether the profiles of coverage depth along the target region differ between the libraries (Fig. 4). To obtain the profiles of coverage depth, segments of the target region were assembled together. Segments within single chromosomes were concatenated according to their coordinates on the chromosome. Chromosomes were concatenated in numerical order, as indicated in the upper horizontal axes. Each point of coverage depth profile represents coverage averaged over 700000 adjacent bases in the concatenated TR (i.e. a total of 101 points in each profile).

The profiles resemble each other closely, particularly the profiles of Standard ES and ThruPLEX-FD ES libraries. Profiles of the GenomePlex ES libraries have greater amplitudes. Visual impression is confirmed by the values of Pearson correlation and average deviation of the profiles relative to the Standard ES profile (Table 4). ThruPLEX-FD ES libraries coverage profile and depth are most close to those of the Standard ES libraries. ThruPLEX-FD ES approach also demonstrates smaller difference between the two test samples than the other protocols.

**Table 4:** Pearson correlation coefficient with coverage profile of Standard ES strategy and average deviation from Standard ES coverage profile.

Strategy	Pearson correlationwith Standard EScoverage profile	Average deviationfrom StandardES coverage profile
ThruPLEX-FD ES, Test DNA1 (DNA2)	0.986 (0.980)	0.318 (0.681)
REPLI-g ES, Test DNA1 (DNA2)	0.809 (0.755)	1.478 (1.950)
GenomePlex ES, Test DNA1 (DNA2)	0.589 (0.947)	3.149 (2.233)

The inter-sample differences observed for the tested protocols might have different reasons. They may be partly caused by the natural variability of a laboratory procedure due to e.g. pipetting and measurement errors. A protocol may be not sufficiently optimized. Also, even a well set up protocol may be sensitive to certain properties of the samples, e.g. purification method used, fragmentation, etc. Small number of samples tested and absence of technical replicas do not allow us to compare the consistency of the approaches.

### Genotype calling

**Figure 5:**
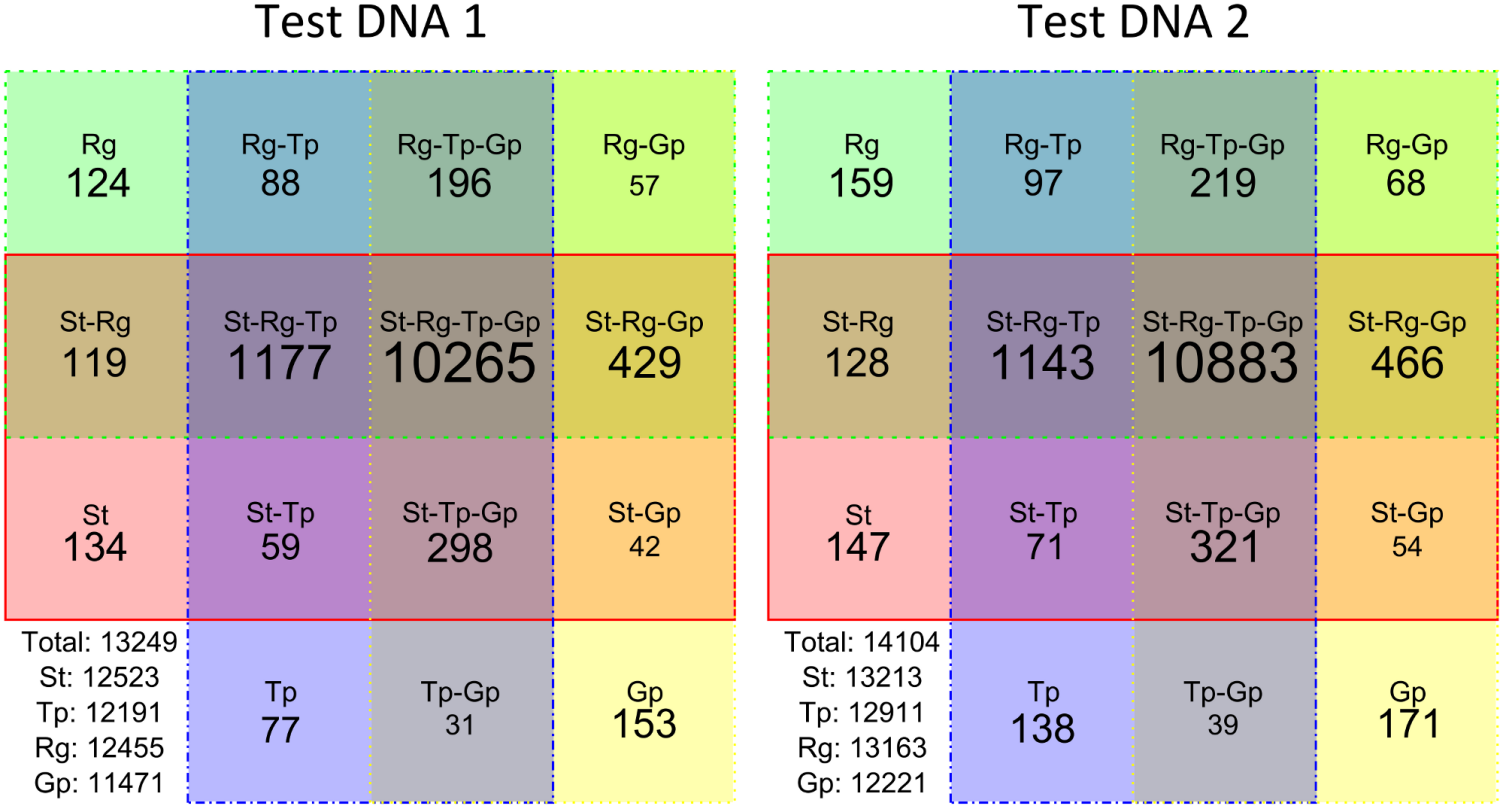
Sharing of genetic variations between strategies depicted in a Venn diagram. Only variation with minimum depth of coverage of 20x and minimum quality of 13 were taken into account in all four strategies. The names of the samples are abbreviated: Standard ES = St; ThruPLEX-FD ES = Tp; REPLI-g ES = Rg; GenomePlex ES = Gp. The lower left tile presents the overall statistics, where “Total” indicates the number of all unique SNVs found in the region of interest, i.e. the union of SNV sets found by each strategy.

Identification of genetic variations is usually the main aim of exome sequencing. We called variations from sequencing data obtained with all tested protocols and compared the results. For the comparison we selected only variations with minimum depth of coverage of 20x and minimum quality of 13 (i.e. the probability of false positive detection is less than 0.05) in all performed strategies. Positions covered with depth ≥20 in all methods constitute 22.1% (23.5%) of the TR for Test DNA 1 (Test DNA 2). Results of this comparison for both Test DNA 1 and Test DNA 2 are presented in Figure 5 in the form of Venn diagram. Each tile represents unique SNVs for a strategy or combination of strategies. For both Test DNA1 and Test DNA 2 all strategies revealed a similar number of SNVs and more than 77% (10265 of 13249; 10883 of 14104) of all detected SNVs are shared by all four methods.

GenomePlex ES libraries reveal more unique SNVs than the other three strategies. The lower right tile in each square diagram (Fig. 5; Gp-tile) represents the number of SNVs that are unique to GenomePlex ES, while Rg- St- and Tp-tiles represent the number of unique SNVs for the other ES strategies. Intersections of GenomePlex ES with other methods contain less SNVs than intersections that do not include GenomePlex ES. However, it is clear that these differences are not significant compared to the number of SNVs shared by all strategies.

Test libraries were also pair-wise compared to the control Standard ES library (Table 5). In all cases there are SNVs detected in just one library in the pair (exclusive SNVs). As a consequence of the higher similarity of coverage profiles of Standard ES and ThruPLEX-FD ES, the part of the target region with ≥20 coverage for both libraries is larger than for other methods. SNV statistics are very similar between ThruPLEX-FD ES and REPLI-g libraries.

**Table 5:** Comparison of SNVs found in regions with coverage > = 20 in both Standard ES and one of three tested strategies.

Strategy	% of TR coveredwith depth≥20for both testedand StandardES libraries	SharedSNVs	Exclusive SNVspresent onlyin StandardES	Exclusive SNVsPresent onlyin tested ES	Discovery rate(found by testedES)/(Total found)
REPLI-g ES, Test DNA1 (DNA2)	34.92 (32.91)	18890 (17561)	1408 (1091)	1281 (1093)	0.9348 (0.9447)
ThruPLEX-FD ES, Test DNA1 (DNA2)	36.95 (37.80)	19836 (19722)	1592 (1803)	1152 (1273)	0.9295 (0.9209)
GenomePlex ES, Test DNA1 (DNA2)	26.27 (29.32)	13010 (14180)	1661 (2226)	803 (789)	0.8927 (0.8705)

Only high-confidence (probability of false positive detection <0.05) SNVs were taken into account.

## Discussion

Hybridization-based enrichment requires a certain concentration of target DNA to provide good hybridization efficiency in a reasonable time. It is also necessary that the library undergoing hybridization is complex enough, and the target region is more or less uniformly represented, otherwise it will not be uniformly represented in the enriched library. Simply increasing the number of PCR cycles for amplifying the pre-capture library does not work, since over-amplified material has a distorted proportion of amplicons relative to initial PCR templates. In addition, highly duplicated library molecules compete with capture probes during hybridization, which leads to a lower output of enriched library, and when capture is performed on a pool of libraries, to under-representation of sequences of low complexity within the pool.

The task of exome sequencing starting from small amounts of DNA can be reformulated as having large enough quantities of sufficiently complex library before hybridization, to obtain enough complex library afterwards. We set out to characterize different protocols designed to address this problem both in terms of exome sequencing and handling of low quantity samples. We assessed the quality of test WES libraries against those using a standard protocol by comparing mapping characteristics, coverage of the target region, and efficiency of SNV detection.

The REPLI-g based protocol turned out to be the best in terms of resulting library complexity, with duplicate levels and mapping parameters being the same as for the standard protocol. The representation of certain genomic regions was distorted differently in the REPLI-g ES libraries compared to the Standard ES libraries (Fig. 4). However from the amount of sequencing data corresponding to ∼20x coverage of the target region, 98% (96.6%) of the target region was represented in REPLI-g ES library samples for Test DNA 1 (Test DNA 2), which is very close to 98.4% (97.5%) in the Standard ES library.

The ThruPLEX-FD library preparation kit looks very interesting, since it closely resembles the procedure for standard library preparation; thus we expected GC amplification biases and sequencing data distribution along the target region to be similar to the standard procedure, as is seen in Figures 3 and 4. The weak point of the ThruPLEX-FD ES library is the lower library complexity, and as a result fewer unique reads map to the target region. However, for the two example samples target region representation was still the same as for the standard ES library, which means library complexity is still within the acceptable range. The less uniform coverage of the target region by the REPLI-g ES library is compensated by the higher percent of reads mapped to the target region than with the ThruPLEX-FD ES library. We ultimately think that both protocols are comparable in the amount of sequencing data required for certain coverage.

The GenomePlex ES libraries showed good complexity. The lower mapping efficiency for the GenomePlex ES strategy is probably related to the presence of the residual WGA-primer sequence in a considerable number of sequencing reads. The most problematic feature of the GenomePlex ES strategy is strong under-representation of GC-rich sequences and uneven coverage of the target region.

In terms of SNV detection, ThruPLEX-FD ES and REPLI-g ES were similar to each other and the standard protocol. GenomePlex ES library sequencing data revealed fewer SNVs and had more exclusive, protocol-specific SNVs. Sequencing data handling was also most problematic for GenomePlex, requiring specific approaches to remove the WGA-primer sequences.

With ThruPLEX-FD, care should be taken when amplifying the pre-capture libraries. It is important to keep the number of cycles as low as possible, and find a balance between obtaining sufficient amounts of material for hybridization, and not over-amplifying to create problems with complexity. In our experience this optimal number of PCR cycles varies between 9 and 11, despite the same amount (10 ng) of starting material. So we recommend following the Rubicon Genomics protocol and performing real-time PCR amplification, in order to follow the amplification curve and remain in the exponential phase.

WGA protocols are safer in terms of material loss, which might be of importance for precious samples. Both REPLI-g and GenomePlex WGA kits reproducibly generated around 5 µg of DNA from 10 ng, providing the possibility to repeat exome library preparation if necessary. In the ThruPLEX-FD protocol, all the material went into the library. Moreover, ThruPLEX-FD requires DNA shearing, which is associated with considerable losses for small amounts of starting material. For example, after fragmentation of 20 ng of genomic DNA using Covaris, only 10 ng are detected afterwards. This is probably related to DNA molecules adhering to the glass walls of the tubes used for shearing. We recommend incubating 0.5x BSA solution in the tubes for several minutes before shearing to reduce DNA binding during shearing.

ThruPLEX-FD ES and GenomePlex ES protocols may be advantageous for partly degraded DNA, since it is recommended that REPLI-g WGA be performed on DNA of good quality.

Apart from the described test experiments we performed REPLI-g ES protocol for three more samples. Sequencing, enrichment and coverage characteristics of the obtained datasets are presented in the Material S1: alignment statistics (Table S1 in Material S1), coverage statistics (Table S2 in Material S1), per-base sequencing depth distribution on the target region (Figure S2 in Material S1), dependence of the coverage on the GC content of the target region (Figure S3 in Material S1) and profiles of coverage depth along the target region (Figure S4 in Material S1). For the altogether five samples the REPLI-g ES protocol demonstrated good consistency.

All the three tested protocols for exome library preparation from 10 ng of starting DNA were successful. Definitely more samples need to be analyzed to make reliable conclusions about the reproducibility of the approaches as well as about superiority of one of them. This study demonstrated that the tested protocols are in general suitable for WES and revealed the parameters which have the tendency to differ between the protocols. GenomePlex ES showed more differences to the standard protocol than REPLI-g ES and ThruPLEX-FD ES protocols. When applying GenomePlex for amplification of original DNA, one should be aware of underrepresentation of GC rich regions in the final library. REPLI-g ES and ThruPLEX-FD ES protocols look like more or less equivalent alternatives, producing sequencing data comparable to the data obtained from 1000 ng of genomic DNA using the standard method. So far REPLI-g ES and ThruPLEX-FD ES protocols seem well-suited for target sequencing library preparation from nanogram amounts of starting material.

## Supporting Information

Figure S1 **Number of sequencing reads obtained per sample in the Agilent SureSelect^XT2^ All Exon assay.** Sample pooling was performed before exome enrichment. Colors mark the protocol used to prepare the pre-capture library. Blue: standard Agilent protocol (data available for the two samples analyzed in this report, Standard ES (1) and Standard ES (2), as well as for 8 other samples), red: REPLI-g ES, green: GenomePlexES, violet: ThruPLEX-FD ES. The original DNA (Test DNA 1 or Test DNA 2) is indicated as a number in brackets.
(TIF)

Material S1 **Three additional human DNA samples were processed according to the REPLI-g ES protocol starting from 10 ng.** This supplement presents the data on sequencing statistics, enrichment efficiency and coverage uniformity for those three samples.
(DOCX)
