# Supplementary figures and images for: Exome Sequencing from Nanogram Amounts of Starting DNA: Comparing Three Approaches
Source: PLoS One. 2014 Jul 3;9(7):e101154. doi: 10.1371/journal.pone.0101154 (PMC4081514; doi:10.1371/journal.pone.0101154)

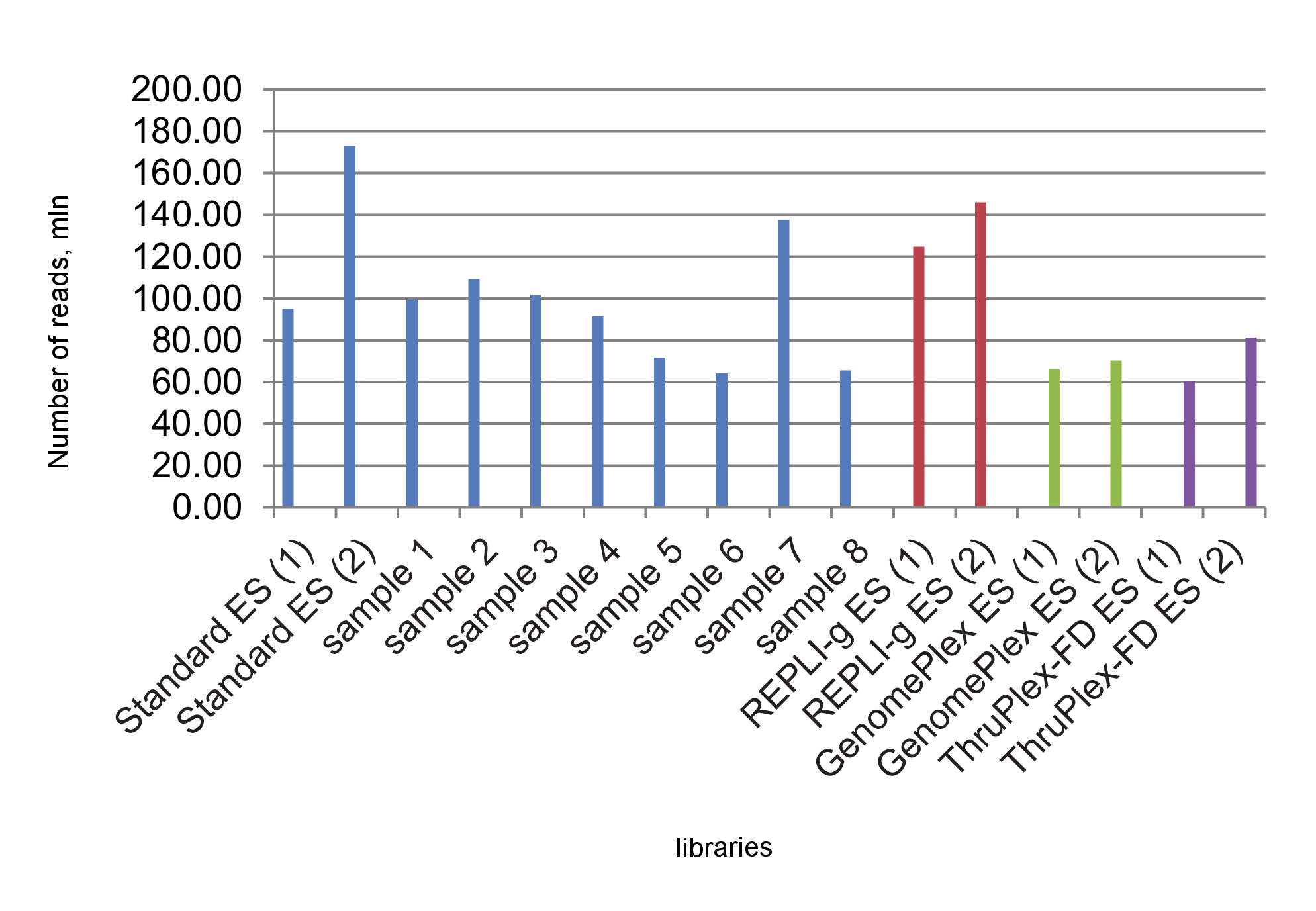

Supplement: Figure S1 — Number of sequencing reads obtained per sample in the Agilent SureSelectXT2 All Exon assay. Sample pooling was performed before exome enrichment. Colors mark the protocol used to prepare the pre-capture library. Blue: standard Agilent protocol (data available for the two samples analyzed in this report, Standard ES (1) and Standard ES (2), as well as for 8 other samples), red: REPLI-g ES, green: GenomePlexES, violet: ThruPLEX-FD ES. The original DNA (Test DNA 1 or Test DNA 2) is indicated as a number in brackets. (TIF) [file pone.0101154.s001.tif]
