# Supplementary material for: Exome Sequencing from Nanogram Amounts of Starting DNA: Comparing Three Approaches
Source: PLoS One. 2014 Jul 3;9(7):e101154. doi: 10.1371/journal.pone.0101154 (PMC4081514; doi:10.1371/journal.pone.0101154)
Supplement: Material S1 — Three additional human DNA samples were processed according to the REPLI-g ES protocol starting from 10 ng. This supplement presents the data on sequencing statistics, enrichment efficiency and coverage uniformity for those three samples. (DOCX) [file pone.0101154.s002.docx]

**Supplementary material S1**

## Performance of REPLI-g ES strategy on three additional samples

In the main text of the paper we characterise three approaches for WES library preparation starting from small amounts of starting material – REPLI-g ES, GenomePlex ES and ThruPLEX-FD ES. Test experiments comparing performance of these three protocols were carried out in parallel on two human genomic DNA samples. Results obtained for these two samples demonstrated that the tested protocols are in general suitable for WES and revealed the parameters which have the tendency to differ between the protocols. However definitely more samples need to be analyzed to make reliable conclusions about the reproducibility of the approaches as well as about superiority of one of them.

Shortly after test experiments were completed we processed three additional samples according to the REPLI-g ES protocol. In this supplement we present data on sequencing statistics, enrichment efficiency and coverage uniformity for those three samples. This data might be supportive for evaluation of the method reliability.

**DNA samples**

The three human genomic DNA samples Sample 1, Sample 2, Sample 3 were isolated from peripheral blood of anonymous blood donors using phenol-chloroform method. As blood sample for Test DNA 2, these samples were collected specifically for the ADAMS FP7 project, mentioned in the Funding section by the group of one of the co-authors, Prof. Evgeny Rogaev. Prof. Rogaev got the approval of the Local Ethical Committee of Vavilov Institute of General Genetics of Russian Academy of Sciences for the ADAMS FP7 project. Prof. Rogaev did not collect blood himself and did not contact the donor, but he has access to the donor-identifying information.

Samples 1-3 were processed at the same time in parallel. The REPLI-g ES protocol was performed as described in the paper, starting from 10 ng of original DNA. Sequencing and data analysis were performed as for Test DNA 1 and Test DNA 2.

Sequencing data for samples 1-3 are submitted to the European Nucleotide Archive (ENA study accession number PRJEB6077).

**Sequencing statistics and target region coverage**

We have performed PEPLI-g ES for all together five samples. For the ease of comparison of Test DNA 1 and 2 and Samples 1-3, data is presented here using the same table and plot formats as in the main paper. Mapping and coverage statistics for Samples 1-3 are presented in Supplementary Table S1 and Supplementary Table S2 respectively. On the figures demonstrating the per-base sequencing depth distribution on the target region (Supplementary Figure S2), dependence of the coverage on the GC content of the target region (Supplementary Figure S3) and profiles of coverage depth along the target region (Supplementary Figure S4) graphs for all five samples processed according to the REPLI-g ES protocol are plotted together.

For Test DNA 1 and Samples 1-3 characteristics of the REPLI-g ES approach are highly consistent. Test DNA 2 results show less similarity to other samples.

Supplementary Table S1. Alignment statistics

| Sample | Number of raw reads (Mb of seq) | Percentage of duplicates (% of raw reads) | Percentage of high-confident reads mapped to hg19 (% of raw reads) | Percentage of high-confident reads mapped uniquely to hg19 (% of raw reads reads) | | | | Percentage of high-confident reads mapped uniquely to FR (% of raw reads) | Percentage of high-confident reads mapped uniquely to TR (% of raw reads) |
| --- | --- | --- | --- | --- | --- | --- | --- | --- | --- |
|  |  |  |  | Total | Mate is mapped (% of Total) | Mate is on the same chromosome (% of Total) | Mate is on the same chromosome and has proper orientation (% of Total) |  |  |
| Sample 1 | 101547324 (10256) | 19.68 | 76.76 | 73.17 | 99.67 | 99.56 | 99.55 | 50.39 | 47.34 |
| Sample 2 | 97122972 (9809) | 19.80 | 76.41 | 72.90 | 99.64 | 99.48 | 99.47 | 51.55 | 48.45 |
| Sample 3 | 106462138 (10752) | 20.14 | 76.08 | 72.39 | 99.63 | 99.46 | 99.46 | 50.09 | 47.86 |

* high confident reads - reads with probability of wrong mapping lower than 0.05 according to their MAPQ score (MAPQ > 13).

** FR - flanking regions (FR), which include 100 bp from both ends of the targeted sequences.

Supplementary Table S2. Coverage statistics for selected high-confident uniquely mapped to TR reads

| Sample | Mean coverage | Coverage depth (% of bases in TR) | | | | | | | |
| --- | --- | --- | --- | --- | --- | --- | --- | --- | --- |
|  |  | 0 | 1 - 10 | 11 - 20 | 21 – 30 | 31 - 40 | 41 - 50 | 51 - 60 | 61+ |
| Sample 1 | 20.73 | 1.65 | 30.32 | 29.14 | 17.79 | 9.69 | 5.10 | 2.67 | 3.50 |
| Sample 2 | 20.80 | 1.56 | 30.75 | 29.95 | 17.49 | 9.12 | 4.70 | 2.49 | 3.83 |
| Sample 3 | 20.94 | 1.76 | 30.03 | 29.35 | 17.75 | 9.57 | 5.02 | 2.66 | 3.75 |

| 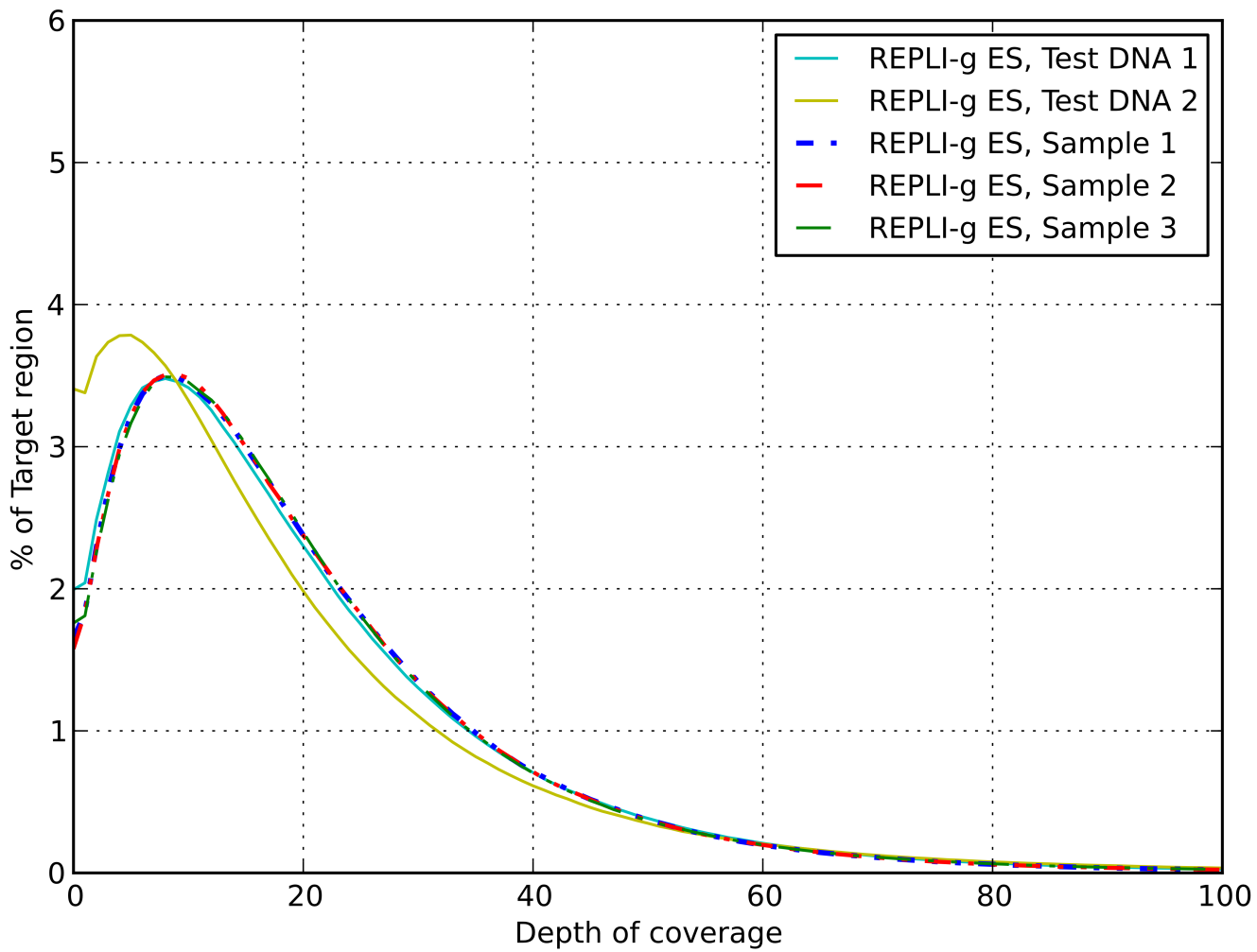 |
| --- |
| **Supplementary Figure S2.** Per-base sequencing depth distribution on the target region. |

`

| 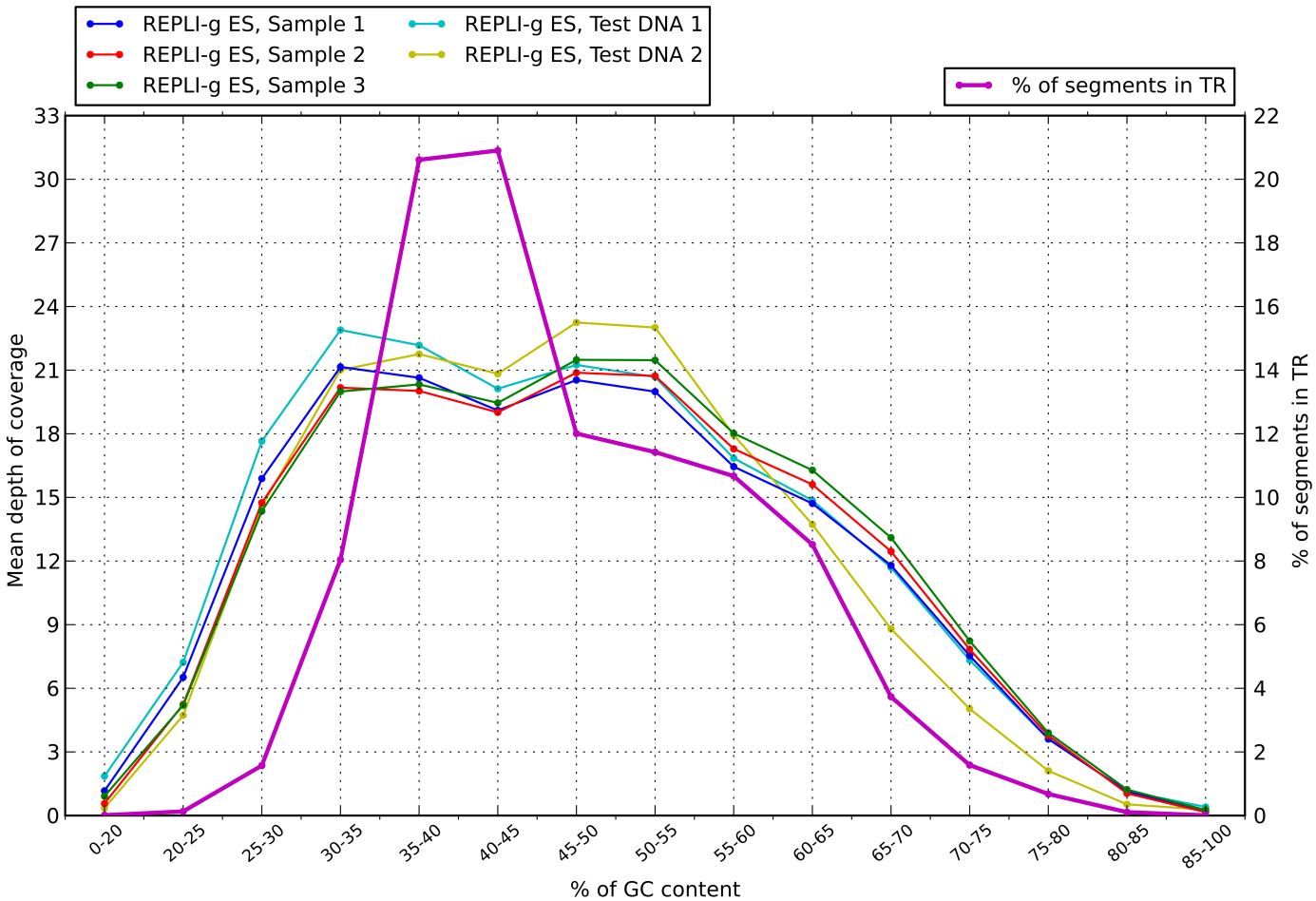 |
| --- |
| **Supplementary Figure S3.** Coverage distribution along the target region segments with different percentages of GC bases. |

| 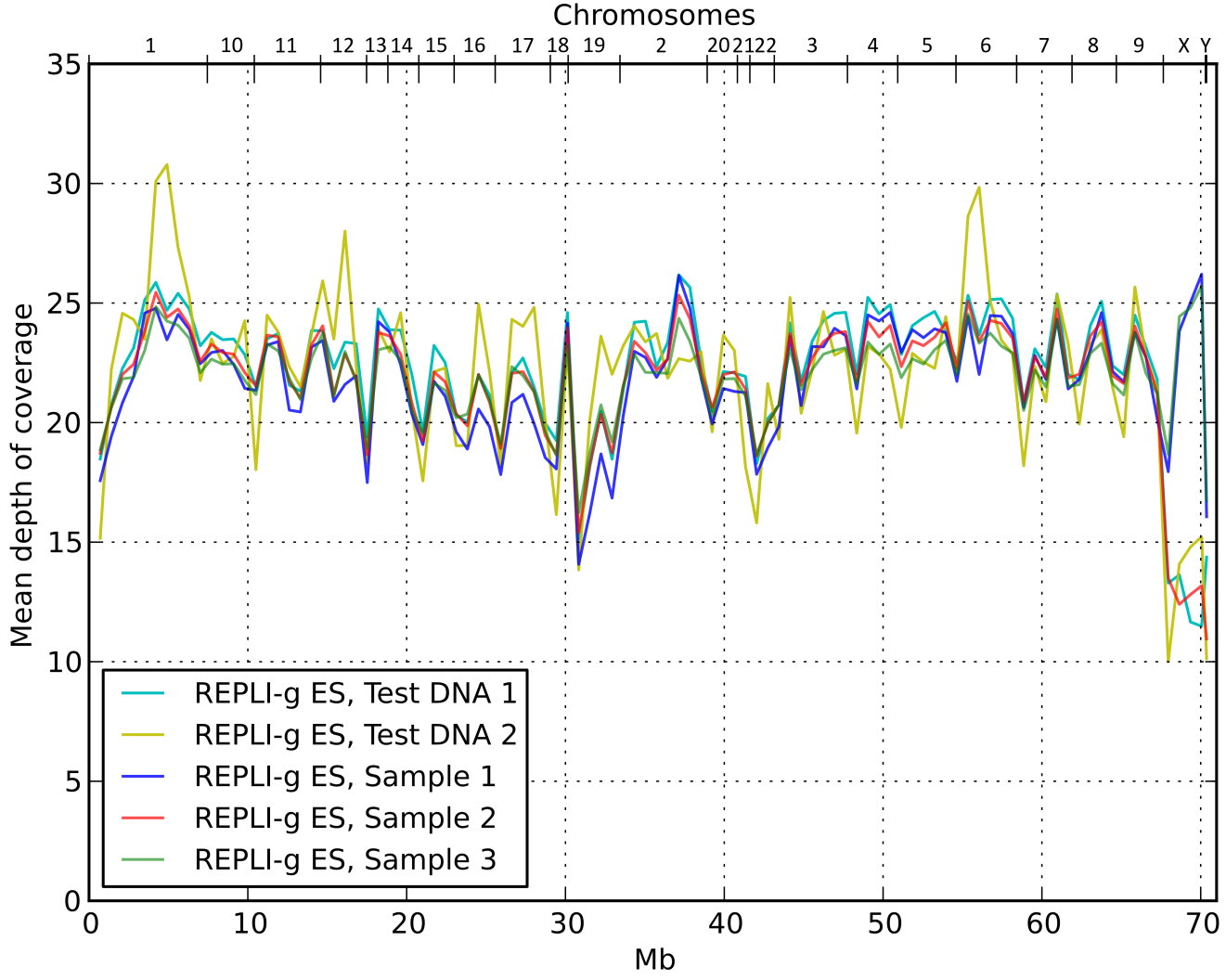 |
| --- |
| **Supplementary Figure S4.** Profiles of coverage depth along the target region. |
